# Understanding Regulatory Mechanisms of Brain Function and Disease through 3D Genome Organization

**DOI:** 10.3390/genes13040586

**Published:** 2022-03-25

**Authors:** Weifang Liu, Wujuan Zhong, Jiawen Chen, Bo Huang, Ming Hu, Yun Li

**Affiliations:** 1 Department of Biostatistics, University of North Carolina at Chapel Hill, Chapel Hill, NC 27599, USA; weifangl@live.unc.edu (W.L.); jiawenn@email.unc.edu (J.C.); 2 Biostatistics and Research Decision Sciences, Merck & Co., Inc., Rahway, NJ 07065, USA; zhongwujuan@gmail.com; 3 Department of Pharmaceutical Chemistry, University of California, San Francisco, CA 94143, USA; bo.huang@ucsf.edu; 4 Chan Zuckerberg Biohub, San Francisco, CA 94158, USA; 5 Department of Biochemistry and Biophysics, University of California, San Francisco, CA 94143, USA; 6 Department of Quantitative Health Sciences, Lerner Research Institute, Cleveland Clinic Foundation, Cleveland, OH 44195, USA; 7 Department of Genetics, University of North Carolina at Chapel Hill, Chapel Hill, NC 27599, USA; 8 Department of Computer Science, University of North Carolina at Chapel Hill, Chapel Hill, NC 27599, USA

**Keywords:** chromosome conformation capture (3C), human genome, brain function and disease, topologically associating domain (TAD), frequently interacting region (FIRE), chromatin interaction

## Abstract

The human genome has a complex and dynamic three-dimensional (3D) organization, which plays a critical role for gene regulation and genome function. The importance of 3D genome organization in brain development and function has been well characterized in a region- and cell-type-specific fashion. Recent technological advances in chromosome conformation capture (3C)-based techniques, imaging approaches, and ligation-free methods, along with computational methods to analyze the data generated, have revealed 3D genome features at different scales in the brain that contribute to our understanding of genetic mechanisms underlying neuropsychiatric diseases and other brain-related traits. In this review, we discuss how these advances aid in the genetic dissection of brain-related traits.

## 1. Introduction

The human genome consists of approximately 3 billion nucleotides, which can form a ~2-meter-long polymer if stretched in one-dimensional (1D) space. However, the average diameter of the nucleus in human cells is ~6 μm. The five orders of magnitude compaction from 1D space to 3D space results in highly complex chromatin spatial organization. How chromatin folds in 3D space have fascinated scientists during the last few decades. Deep understanding of the principles of chromatin folding holds great promise to reveal the structural basis of gene regulation and genome function [1,2,3,4,5,6,7].

To achieve this goal, genome-wide experimental assays are essential to accurately characterize the 3D genome in the nucleus. Harnessing the power of next generation sequencing technologies, high-throughput chromatin conformation capture (Hi-C) [8] has been widely applied to cultured cell lines, purified cell types, and complex tissues [9,10,11], and has revealed 3D genome features at a cascade of resolutions. Specifically, at chromosome resolution, different chromosomes occupy distinct locations in the nucleus, termed as chromosome territories (CTs) [12], where transcriptionally active regions are near the nuclear center, while transcriptionally inactive regions are near the nuclear periphery and tend to be associated with the nuclear lamina. Zooming in, each chromosome consists of mega-base (Mb) resolution A/B compartments [8] and sub-compartments [9], with more frequent interactions within the same type of compartment, and infrequent interactions between the two different types of compartments. The compartments can be further divided into topologically associating domains (TADs) [13,14], which are typically several hundred kilobase to ~1 Mb in size. TADs function as the basic building block of the 3D genome, dictating the majority of chromatin interactions to be within the same TAD. Moving to finer tens of kilobase resolution, we have identified frequently interacting regions (FIREs) that mark regions of the genome and have a high level of interactions with their neighboring regions [11]. Finally, at the finest kilobase resolution, two types of chromatin loops have been discovered, mostly inside of TADs. One type is structural loops, which are mediated by the convergent CTCF motif pairs and largely conserved among different cell types [9]. The other type is functional loops, which are formed by enhancer–promoter interactions and exhibit high cell-type specificity [15,16,17]. Extensive studies have demonstrated that these 3D genome features are closely related to transcriptional regulation mechanisms, the determination of cell identity, and organism-level health and disease outcomes [18,19,20]. 

Although extremely successful, Hi-C technology has two key limitations. First of all, Hi-C requires high sequencing depth to profile genome-wide chromatin organization features, in particular for mapping kilobase resolution chromatin loops, making it cost-prohibitive for large scale studies. Several more cost-efficient experimental approaches have been developed to measure genome-wide chromatin interactions at pre-selected loci of interest (e.g., capture Hi-C [21]), or protein-mediated chromatin interactions (e.g., ChIA-PET [22], PLAC-seq [23], and HiChIP). In addition, Hi-C relies on proximity ligation to quantify pair-wise chromatin contact frequency among the cell population. It therefore has limited sensitivity to capture complex chromatin folding events that involve more than two DNA segments. As orthogonal approaches, ligation-free technologies, including GAM [24,25] and SPRITE [26], can achieve high sensitivity of profiling ultra-long-range (>10 Mb) intra-chromosomal interactions, inter-chromosomal interactions, and multi-way interactions. In addition, imaging-based technologies have been developed and leveraged to study dynamic genome structure with ever increasing temporal and spatial resolutions. Excellent reviews are available for readers interested in the advancement and applications of imaging-based technologies [27,28,29,30,31,32].

All above-mentioned genomics technologies are designed to measure chromatin spatial organization in a population of cells. Recent rapid advances in single cell genomics technologies enable us to profile 3D genome structure in single cells. Depending on the trade-off between throughput and coverage, one can apply single cell Hi-C [33,34], single nucleus Hi-C [35], or Dip-C [36] to assay thousands of cells with on average ~1 million contacts per cell, or single-cell combinatorial index Hi-C (sci-Hi-C) [37,38,39] to assay a larger number of cells (e.g., over tens of thousands of cells) with shallower coverage (e.g., on average a few thousand contacts per cell). Another promising technology is co-assay of 3D genome and epigenome in the same cell, such as sc-m3c-seq [40,41] and single cell methyl-Hi-C [42]. Applying these single cell 3D genomic technologies can reveal cell-to-cell variability of the 3D genome among homogenous cell populations, and more importantly, discover cell-type-specific 3D genome features obscured by complex heterogeneous tissue samples.

With the fast development of Hi-C and Hi-C-derived technologies and rapid accumulation of chromatin interactome datasets, a variety of computational methods have been proposed to analyze such data and have revealed multi-scale 3D genome features, including A/B compartments and sub-compartments, TADs, FIREs, structural loops, and enhancer–promoter interactions. Meanwhile, recent efforts have been spent on developing novel computational methods tailored for single cell Hi-C datasets, which can identify A/B compartments, TAD-like structures, and chromatin loops from single cells [43,44,45]. Comprehensive description of computational methods for 3D genome in bulk cells and single cells can be found in recent review articles [46,47,48]. 

## 2. Human Brain Genome Organization and Its Relevance to Neuropsychiatric Disorders

Human brain, a central organ of the human nervous system, is a highly complex organ that regulates many essential processes including cognition, memory, emotion, vision, breathing, motor skills, and experiences of surroundings [49]. As the most complex organ in the human body, the brain manifests its complexity in various aspects. First, the human brain exhibits complicated spatial organization [50]. Specifically, it has the six-layered cerebral cortex, shared with other mammals, but notably larger in size. Underneath the cerebral cortex, there are many indispensable structures encompassing the thalamus, the epithalamus, the striatum, the pineal gland, the pituitary gland, the hypothalamus, the subthalamus, the substantia nigra, as well as the limbic structures, including the amygdala and the hippocampus. A number of studies [51,52,53,54,55], particularly through examining gene expression and epigenetic profiles from various regions of the brain, have identified the most associated regions for different brain-related disorders. For example, schizophrenia (SCZ), intelligence, educational attainment, neuroticism, and major depressive disorder (MDD) have been found to be most significantly associated with the cortical regions; Parkinson’s disease was found to be most strongly associated with the expected substantia nigra; while Alzheimer’s disease (AD) shows consistent association with tissues playing prominent immune-related roles from multiple studies [53,54,55]. 

Besides the numerous anatomical structures aforementioned, the human brain consists of diverse cell types. Specifically, there are two major categories of cell types in the brain, namely, neuronal cells and glial cells, as well as other cell types including vascular cells (such as pericytes) and endothelial cells [56,57,58]. The glial cells can be further divided into astrocytes, oligodendrocytes, and microglia [59]. Neuronal cells encompass an extraordinary diversity and can be further divided into dozens of subtypes under the two major cell subtypes: excitatory neurons and inhibitory neurons [57,60]. As gene regulation varies substantially across cell types, and relevant cell types differ for different diseases and traits, it is important to study chromatin spatial organization across diverse cell types and understand gene regulation mechanisms in a cell-type-specific manner. Some recent efforts have been made, including the interrogation of neuronal cell types derived from induced pluripotent stem cells, and also of primary cells obtained through cell sorting including different types of neurons, astrocytes, microglia and oligodendrocytes [61,62,63,64].

Furthermore, the brain is also temporally complex. For example, the development of the nervous system, commonly termed corticogenesis, is a highly complex process that requires the balancing of many components, including chromatin spatial organization. Disentangling the interplay of these contributing components is critical to the understanding of various diseases associated with dysfunctional cortical development, as demonstrated in Song et al. [63]. Several other studies have also interrogated the relevance of varying developmental stages, primarily the fetal and adult brain [62,65,66,67], to shed insights into the temporal dynamics underlying genetic regulation, and ultimate disease and health-related outcomes. 

**Figure 1:**
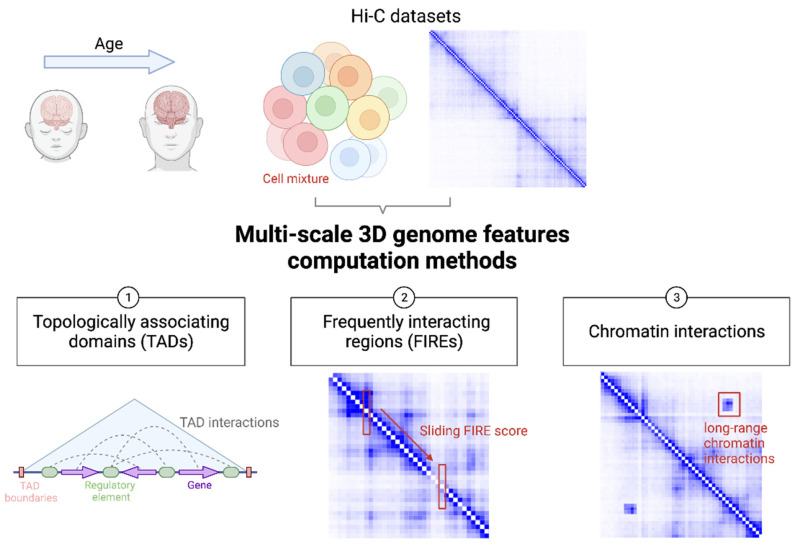
Cartoon illustration of utilizing brain 3D genome organization data at multiple scales to understand genetic mechanisms of brain function and disease. Hi-C and like datasets can be generated from brain samples from donors at different ages. Typically, each sample contains a mixture of different brain cell types. From a typical Hi-C dataset, TADs, FIREs, and chromatin interactions can be defined at different developmental stages and from distinct cell types for comparative analysis.

Figure 1 illustrates state-of-the-art strategies to harness the power of multi-scale readouts from brain 3D genome organization data for the understanding of genetic mechanisms underlying neuropsychiatric diseases and other brain-related traits. In the sections below, we will showcase how TADs, FIREs, and chromatin interactions can aid in the genetic dissection of brain-related traits. 

### 2.1. TAD

TADs have been largely under-appreciated and under-utilized in the interpretation of genetic findings for brain-related disorders and traits, mainly because of two reasons. First, TADs, as a stable structural feature, are rather conserved across tissues, cell lines, and even across species [13]. Second, rare structural variants (SVs) that are more likely to result in abnormal TAD formation are usually not available for analysis because prevailing genotyping arrays and short read sequencing technologies have limited capabilities to generate reliable genotypes for SVs.

Here, we use SCZ as an example. As a heritable disease, SCZ has been extensively studied via genotyping-array-based GWAS [68,69,70], as well as via whole exome sequencing (WES) [71] and whole genome sequencing (WGS) [72]. However, WES only analyzes the protein-coding portion of the genome (around 3%), and therefore misses most regulatory regions. Previous findings [73,74] indicate rarer and evolutionarily younger SNPs tend to have higher SNP heritability for numerous complex traits. The interrogation of the rarer regulatory variants entails WGS-based studies, which allow nucleotide-level resolution profiling of the entire genome including the vast non-coding regions. In addition, WGS empowers the detection of SVs throughout the accessible genome.

Halvorsen et al. examined the role of genetic variations that can be discovered by WGS but not by standard genotyping array or WES in the SCZ etiology [72]. A higher genome-wide load of rare SVs including deletions (DEL), tandem duplication (DUP), inversion (INV), and mobile element insertion (MEI) sites has been identified in SCZ cases than in controls. Burden analyses of ultra-rare SVs further revealed that ultra-rare DELs are highly enriched in SCZ cases, while ultra-rare DUP and INV are neither significantly enriched nor depleted.

**Figure 2:**
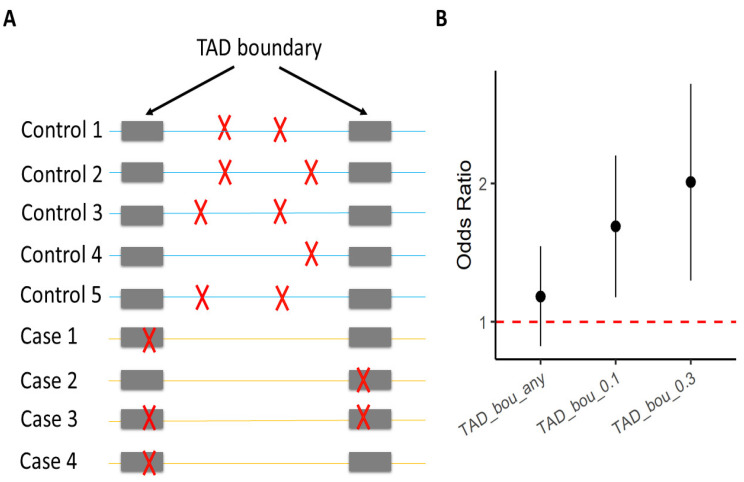
Enrichment of ultra-rare SVs in SCZ cases that impact TAD boundaries. The figure was inspired by results presented in Halvorsen et al. [72]. (**A**) Each row represents one individual; gray bars indicate TAD boundaries; red crosses mark ultra-rare SVs. (**B**) The Y-axis is the odds ratio that measures the increase in the likelihood of being a SCZ case per unit increase in burden of ultra-rare SVs [72]. The X-axis specifies different sets of ultra-rare SVs on which the burden analyses were performed. “TAD_bou_any”: ultra-rare SVs that have (≥1 bp) overlap with TAD boundaries identified from the adult brain; “TAD_bou_0.n”: ultra-rare SVs that overlap > n × 10% of TAD boundaries identified from the adult brain.

TAD boundary disruption by SVs has been linked to several developmental disorders [75,76]. To help elucidate the molecular mechanisms of the enrichment of ultra-rare DELs among SCZ cases, Halvorsen et al. partitioned the elevated genome-wide burden of ultra-rare SVs among various functional elements including TADs, FIREs, ATAC-seq peaks, CTCF ChIP-seq peaks, H3K27ac ChIP-seq peaks, and H3K4me3 ChIP-seq peaks. They found that only TAD boundaries are significantly enriched with these ultra-rare SVs (Figure 2). Specifically, their results suggest that ultra-rare SVs in SCZ cases disrupt TAD boundaries, detected from both the adult brain and the fetal brain. Modifying TAD boundaries can substantially influence enhancer–promoter interactions, and disrupt local gene expression [75,76]. 

Overall, the Halvorsen et al. study provides an exemplary case where TADs are systematically assessed for their SCZ relevance. This example highlights the importance of TADs in the understanding of genetic variation linked with brain-related diseases.

### 2.2. FIRE

By analyzing a compendium of Hi-C data generated across 21 human cell lines and primary tissues, Schmitt et al. [11] discovered FIREs as local interaction hotspots enriched for active enhancers. Crowley et al. [66] further implemented the Poisson regression-based approach in a stand-alone computationally efficient R package FIREcaller to identify FIREs.

**Figure 3:**
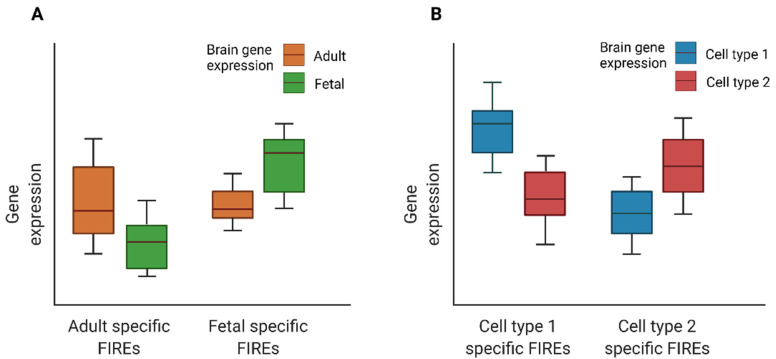
Cartoon illustration of expression for genes overlapping developmental time- or cell type-specific FIREs: (**A**) Boxplots of expression for genes overlapping fetal or adult brain FIREs. (**B**) Boxplots of expression for genes overlapping cell type specific FIREs. The figure was inspired by results presented in Schmitt et al. [11] and Crowley et al. [66].

FIREs are chromatin features distinct from other 3D genome features such as A/B compartments, TADs, and chromatin loops, in terms of high cell type specificity. Schmitt et al., among the 21 tissues mentioned before, found that roughly 38.8% of FIREs were found in only one tissue or cell type, and about 57.7% were found in two or fewer, indicating that FIREs are highly tissue specific [11]. FIREs are enriched in compartment A and depleted in compartment B. In addition, FIREs are depleted near TAD boundaries but are enriched within TAD and towards TAD centers. Although FIREs are enriched for chromatin loop anchors, 90% of FIREs are within chromatin loops. The dynamics of FIREs across brain developmental stages and cell types are closely associated with gene regulation dynamics during brain development and in different cell types [64,66] (as illustrated in Figure 3).

Disease candidate genes can be prioritized by examining genes near FIREs containing disease-associated GWAS SNPs [11]. Then, by investigating the overlap between FIREs and disease-associated SNPs, Schmitt et al. found that FIREs are enriched for disease-associated GWAS SNPs [11]. For example, they reported that an AD-associated SNP rs3851179 resides in a hippocampus-specific FIRE. Examining genes nearby, they found a putative causal gene for AD: gene *PICALM*, whose 5′ end overlaps this brain-specific FIRE and is 88.5 kb away from this SNP. Another example, as shown in Crowley et al. [66], is a SCZ-associated GWAS SNP rs9960767 residing in a hippocampus super-FIRE, which overlaps with two hippocampus super-enhancers. The gene *TCF4*, to which this SNP rs9960767 is intronic, is a potential causal gene for SCZ. The hippocampus super-FIRE region within its gene body also helps to suggest the underlying regulatory mechanism.

FIREs are genomic regions that are involved in gene regulation. In a recent study, Hu et al. compared FIREs in sorted NeuN+ (representing neurons) and NeuN− (representing glia) cells to identify differential FIREs [64]. Specifically, they defined NeuN+ specific FIREs as regions with higher FIRE scores in NeuN+ cells but lower FIRE scores in NeuN− cells; and NeuN− specific FIREs are conversely defined. Their results suggest that most NeuN+ or NeuN− specific FIREs overlap with the corresponding NeuN+ or NeuN− specific H3K27ac ChIP-seq peaks. Furthermore, genes associated with NeuN+ and NeuN− specific FIREs are primarily enriched in neurons and glial cells, respectively. These findings revealed that differentiated FIREs in the central nervous system are closely linked to cell-type-specific gene regulation. Furthermore, NeuN+ hypoacetylated and NeuN− hyperacetylated genes are enriched in co-expression modules that were downregulated and upregulated in AD, respectively. Taken together, these results suggest that FIRE-associated cell-type-specific gene regulatory networks can aid in the understanding of AD etiology. 

### 2.3. Chromatin Interactions

The disruption of regulatory chromatin loops plays an important role in the etiology of complex brain disorders. For example, several studies [43,63,77,78,79,80,81,82] have reported non-coding variants identified from genome-wide association studies (GWAS) overlapping with cis-regulatory elements that regulate distal genes by long-range chromatin interactions. It remains challenging to identify causal variants and their putative target genes in the disease relevant cell types due to the complexity of the brain tissue and the etiology underlying brain-related disorders.

Assigning variants to genes based 1D proximity provides a limited, if not sometimes misleading, view of the complexity of GWAS findings. Integrating chromatin interactome data with GWAS results can aid in the identification of potential causal variants, their effector genes, and the functional roles at each GWAS locus. For example, a recent study on AD utilized Hi-C data from fetal and adult brains to examine possible mechanisms that contribute to the regulatory effects of risk haplotypes at the *APOE* locus (encompassing multiple genes including *PVRL2*, *APOE*, and *APOC1*) on the expression of nearby genes in brain tissues [83]. They identified multiple highly interacting regions covering the risk haplotypes, suggesting broad modulatory effects of those non-coding haplotypes beyond the widely known *APOE* gene at the locus. In addition, a recent SCZ study leveraged Hi-C data from the developing brain to aid in the identification of putative causal SNPs by mapping SNPs to regions identified as likely regulatory elements [69]. Moreover, Giusti-Rodriguez et al. mapped GWAS loci associated with ten psychiatric disorders and cognitive traits, including SCZ, intelligence, attention deficit hyperactivity disorder (ADHD), alcohol dependence, AD, anorexia nervosa, autism spectrum disorder, bipolar disorder (BD), major depression disorder, and educational attainment, to thousands of genes by leveraging Hi-C data from adult and fetal brain cortex samples with concomitant RNA-seq, open chromatin (ATAC-seq), and ChIP-seq data (H3K27ac, H3K4me3, and CTCF). Linking GWAS variants to their potential effector genes helps the interpretation of identified genetic associations for these complex brain-related diseases and traits in non-coding regions [67].

Disease-associated variants are often found in cell-type-specific enhancers, which form regulatory interactions with the promoter regions of their target genes [71]. With the development of single cell technologies, candidate target genes can be assigned to non-coding GWAS SNPs in a cell-type-specific manner. For example, Yu et al. [43] recently developed SnapHiC, a computational method to identify chromatin interactions from single cell Hi-C data, and leveraged cell-type-specific chromatin interactions to find putative target genes, which are likely regulated by GWAS variants associated with neuropsychiatric disorders in disease relevant cell types. 

**Figure 4:**
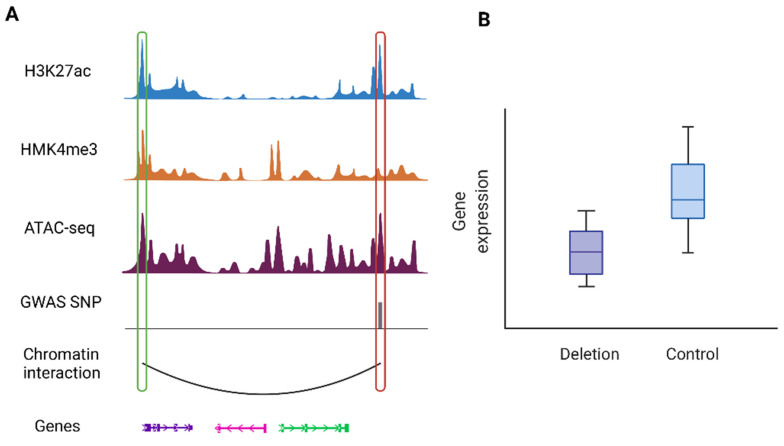
An illustrative example of a chromatin interaction involving disease-associated variants: (**A**) An example of a PIR containing a risk variant (highlighted by the red box on the right) with promoter region of the target gene (highlighted by the green box on the left). (**B**) Regulatory roles of the risk variant can be validated by downstream experiments such as CRISPR techniques. This example shows deletion of the PIR containing the risk variant results in downregulation of the target gene (the left most gene). The figure was inspired by results presented in Song et al. [61].

Besides Hi-C, technologies such as promoter capture Hi-C and PLAC-seq can also help elucidate genes for neuropsychiatric disorders. Song et al. found that GWAS SNPs are enriched at promoter interacting regions (PIR) in a disease- and cell-type-specific manner [61]. Specifically, the study generated promoter capture Hi-C data for primary astrocytes and three neuronal cell types derived from induced pluripotent stem cells, from which chromatin interactions were identified in a cell-type-specific manner. They then leveraged these cell-type-specific chromatin interactions to annotate genetic variants associated with eleven complex neuropsychiatric disorders. Results showed that ASD, mental process (MP), and SCZ SNPs are enriched at PIRs across all cell types. Unipolar depression (UD) SNPs are enriched exclusively in excitatory and hippocampal dentate gyrus (DG)-like neurons, whereas AD, ADHD, and BD SNPs also exhibit enrichment in lower motor neurons. The regulatory roles of PIRs were further validated by CRISPRi experiments [61]. Figure 4 shows an illustration of such cases where a PIR containing a disease risk variant interacts with the promoter of its target gene to regulates gene expression. Likewise, analysis of human post-mortem cortical tissue shows that sporadic AD variants are largely involved in microglia-specific chromatin interactions, while variants associated with various neuropsychiatric disorders are primarily confined to neuronal-specific enhancer-promoter networks [62].

We have focused mainly on regulatory interactions involving single nucleotide polymorphisms (SNPs). Few studies have investigated the impact of larger-scale or more complex rearrangements of DNA sequences on chromatin interactions because these rearrangements are much less well characterized than SNPs [84,85]. In the earlier TAD section, we reviewed the Halvorsen et al. study, which showed the impact of rare SVs on TADs. Recent studies have started to assess the effect of SVs on chromatin interactions. For example, Johnston et al. [86] observed many chromatin interactions involving DNA segments >1 Mb apart or even >100 Mb, in their Hi-C data from glioblastoma stem cells. Hypothesizing that SVs may explain these surprisingly long-range interactions, they explicitly studied interactions involving SVs or not, finding that the apparent distance of interactions involving SVs is >10×that of interactions without SVs in the neighborhood. They presented a representative example at the *JAK1* locus where a 140 Mb inversion together with other large deletions moved two enhancers residing normally on the q-arm of chromosome 1 to the p-arm near the *JAK1* gene, leading to chromatin interactions that would be normally impossible. Similarly, Wang et al. [87] reported enhancer hijacking that resulted from SVs in their Hi-C data from cell lines derived from patients affected with pediatric high-grade gliomas. 

## 3. Integrative Omics Analysis 

Genome spatial organization has a complex interplay with various other molecular machineries, including variants in the DNA sequence (such as SNPs, copy number variants, and general structural variants), epigenomic modification (DNA methylation and histone modification), and transcription factor binding. In brain, these factors together orchestrate the expression of genes, shape gene co-expression networks, affect molecular phenotypes (such as neuron variability, autophagy progression, endosomal trafficking, microglia phagocytic activity, etc.), to eventually exert their influence on ultimate organism level phenotypes manifested as brain-related traits or disorders. Therefore, integrative analysis to leverage multi-omics data along with chromatin conformation information has been delivering on the promise of revealing biological insights that would be missed by separately analyzing individual data modality, and thereby generating more robust and trustworthy mechanistic hypotheses. Many investigators have made efforts to jointly analyze multi-omics data [88,89].

Integrative analysis can be the simple joint consideration of data from domains other than genome structure without introducing complicated computational models. For example, to interpret AD GWAS variants, Nott et al. considered enhancer information besides enhancer–promoter interactions revealed from H3K4me3 PLAC-seq data, and constructed cell-type-specific gene-networks by performing protein–protein interaction network analysis based on the effector genes suggested from enhancer and chromatin interactome information [62]. Using this simple yet straightforward integrative strategy, they identified a microglia-specific enhancer region harboring AD-associated GWAS variants and validated its regulatory role on the *BIN1* gene through CRISPR/Cas9 experiments in microglia, but not in neurons or astrocytes. Similar simultaneous consideration of other omics data together with chromatin conformation information can be found in other studies [61,63,64,77]. For example, Song et al., conducted linkage disequilibrium score regression (LDSC) analysis to partition the heritability of genetic variants residing in putative cell-type-specific enhancer regions (jointly defined by PLAC-seq and ATAC-seq data) in four fetal neuronal cell types to prioritize the most relevant cell types for various neuropsychiatric disorders [63].

Other studies involve explicit modeling or more complicated integrative analysis. For example, Fulco et al. [90] proposed the activity-by-contact (ABC) model to qualify the connection between a putative regulatory element and its target gene. Specifically, an ABC score is calculated from three quantities: the element’s “activity” level, the intensity of chromatin interaction between the element and its target gene, and the relative effect of the element on the gene. Such a model is motivated by the biochemical forces determining the effect of a regulatory element on its effector gene and aids in the quantitative ranking of element–gene pairs. Nasser et al. [91] applied the ABC model to create a genome-wide enhancer maps in 131 human cell types and tissues to link GWAS variants to disease genes. In contrast, the H-MAGMA method [92] involves more complex integration of information from multiple domains, to unveil neurobiological mechanisms underlying brain disorders. Specifically, H-MAGMA first combined chromatin spatial organization information from brain tissues with SNP annotations to assign SNPs to genes, then performed gene-based association analysis by further incorporating GWAS results for brain disorders, and finally provided insights into the biological mechanisms in four different forms including genetic correlation among the disorders, curve to suggest the developmental stage most relevant to the disorders, disorder-specific ranking of the most relevant cell types, and pathway enrichment analysis for each disorder. Similarly, Li et al. [65] performed integrative genomics analysis encompassing multiple domains of brain-related functional information across developmental stages for the better understanding of neuropsychiatric disorders. The authors compiled a comprehensive variety of genomic, regulatory, epigenomic, and transcriptomic features of the human brain across cell types, brain regions, and developmental stages. The compiled diverse modalities were jointly modeled with the WGCNA [93] gene co-expression network analysis to reveal gene modules (or “module eigengenes”) for various brain-based traits and disorders. Most identified gene modules exhibited spatiotemporal or temporal specificity and were enriched for gene expression associated with distinct cell types.

Integrative analysis involving chromatin spatial organization data is still in its infancy. These aforementioned studies highlight some first attempts. Innovative integrative methods are much needed to maximally extract information underlying the complex molecular interplay in the brain. 

## 4. Single Cell Analysis

**Figure 5:**
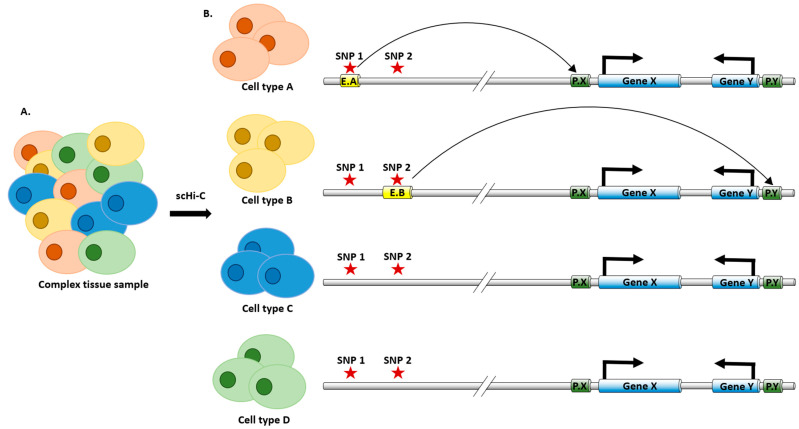
A cartoon illustration of the cell-type-specific enhancer–promoter interactions linking GWAS variants to their putative target genes: (**A**) The complex tissue consists of four cell types (A, B, C, and D), where cell types A (red) and B (yellow) are disease relevant, cell types C (blue), and D (green) are not disease relevant. (**B**) Two GWAS variants SNP1 and SNP 2 (highlighted by the red star) locate upstream of gene X and downstream of gene Y (genes are shown as blue cylinders). In cell type A, SNP 1 resides in cell-type-A-specific enhancer E.A (highlighted by the yellow cylinder), interacting with the promoter of gene X (P.X, highlighted by the green cylinder). In cell type B, SNP 2 resides in cell-type-B-specific enhancer E.B (highlighted by the yellow cylinder), interacting with the promoter of gene Y (P.Y, highlighted by the green cylinder). In cell types C and D, neither SNP 1 and nor SNP 2 resides in enhancers, and there is no chromatin interaction between GWAS SNPs and gene promoters. In this cartoon illustration, gene X is the putative target of GWAS SNP 1 in cell type A, while gene Y is the putative target of GWAS SNP 2 in cell type B.

Most of the existing genome-wide chromatin spatial organization data for the brain were derived from bulk tissue samples, easily masking or obscuring cell-type-specific profiles in brain samples consisting of many distinct cell types. Single cell Hi-C data (scHi-C) have gained increasing popularity in recent years, thanks to advances in both experimental technologies and computational approaches [47,48]. We now have scHi-C data containing thousands of brain cells with up to one million contacts per cell [40,94]. Data sparsity, however, remains a grand challenge in the analysis of scHi-C data because these single cells, even when aggregated within each major cell type, remain much shallower in their total number of contacts for reliable and powerful identification of chromatin interactions. Recent methods based on data augmentation have shown promising results [36,43,44,45]. These methods adopt various data augmentation approaches, including contact imputation [43,45], hypergraph construction [44], and interpolation based on haplotype-resolved 3D models constructed [36]. For example, Yu et al. [43] proposed the SnapHiC method, which identifies chromatin loops from scHi-C data by first applying the random walk with the restart algorithm [45] for each single cell, and then aggregating normalized contacts after imputation across cells of the same cell type to detect loops. The authors applied the SnapHiC method to single-nucleus methyl-3C-seq data from 2,869 human prefrontal cortical cells [40] and identified chromatin loops specific to several neuronal, and non-neuronal types. These identified cell-type-specific enhancer–promoter interactions connect non-coding GWAS variants with their effector genes in a cell-type-specific manner (Figure 5). For example, two AD-associated GWAS variants exert their function on the *APOE* gene in astrocytes, supported by their astrocyte-specific chromatin loops.

Although promising, particularly with continuous technological advances and increasingly accumulated data, scHi-C data entail the development of rigorous and powerful computational methods. Besides the detection of pairwise chromatin loops discussed above, scHi-C data can potentially reveal various other types of genome structure features, including multi-way chromatin interactions, A/B compartments, and TAD-like structures. With larger number of cells interrogated, and each cell with at least hundreds of thousands of contacts, we anticipate future analysis to further reveal biological variation across single cells from the same cell type, which has been largely unexplored due to data sparsity. In addition, joint analysis of scHi-C data with bulk data can also allow us to take advantages of both types of data. For example, Rowland et al. [95] identified expected cell-type-specific spatial organization profiles by deconvolving bulk Hi-C data from brain cortex. Naïve application of MUSIC [96] developed for RNA-seq data showed the advantage of integrative analysis with the matched scHi-C data. Similar methods tailored specifically for Hi-C data can further empower investigators with a mixture of bulk and single cell Hi-C data. In addition, co-assaying Hi-C and other epigenomic features within the same single cell, enabled by recently developed technologies such as Methyl-HiC [42] and methyl-3C [40,41], are expected to advance our understanding of the relationships and dynamics of genome structure with other molecular profiles at single cell resolution. Finally, orthogonal technologies including ligation-free technologies such as immunoGAM [25] and imaging-based technologies such as DNA seqFISH+ [97] have also been harnessed to interrogate chromatin spatial organization in mammalian brain tissues. 

We have so far focused on single cell or single nucleus sequencing-based technologies and computational methods developed for data generated from these technologies. Recent years have also witnessed tremendous advancements in imaging-based technologies to map the spatial arrangement and interaction of chromatin in the nucleus. Table 1 provides a brief summary of the main characteristics of imaging- and sequencing-based technologies, focusing on those that achieve single cell resolution. Interested readers can refer to most recent review papers [47,48,98] to learn more about these technologies and computational methods tailored to them.

## 5. Discussion

In the past decade, we have made great strides in generating 3D genome structure data in the brain tissue and their contributing cell types. We have also made great progress in employing these data to decipher the molecular codes underlying brain-related diseases and traits. Specifically, the extensive literature provides mature computational methods and pipelines to generate multi-scale readouts of 3D genome structure information from Hi-C and Hi-C-derived technologies. These readouts include A/B compartments, TADs, FIREs, and functional and structural chromatin loops or interactions, particularly in bulk samples and burgeoning in single cells. In this paper, we focus on reviewing what major resources have been generated (section “Relevant resources”) and how these datasets have been harnessed to provide insights for the molecular understanding of various brain-related traits and disorders. Such insights can also help us prioritize follow-up experiments, including CRISPR and CRIPSRi genome or epigenome editing experiments followed by bulk or single cell RNA-sequencing to validate the effect of putative regulatory elements on their target genes [63,104,105,106]; and gene perturbation experiments such as shRNA [107,108] to assess the further downstream analysis in molecular or cellular phenotypes. 

Multiple other directions have been explored but not covered in this review. First, for chromatin loops, we have focused on pairwise interactions. It is appreciated that multi-way interactions can help elucidate chromatin hubs consisting of multiple genes and/or regulator elements [24,25,26]. We expect more research in this direction with more technological and computational methods developed [24,26,109]. Second, little research has been carried out to interrogate inter-chromosomal interactions, due to the sparsity of contacts between DNA segments from different chromosomes. However, existing literature has shown evidence underscoring the relevance and importance of inter-chromosomal contacts affecting brain-related phenotypes including intellectual ability and autistic behavior [110,111,112]. We hope future studies present ways to enhance our capabilities to study inter-chromosomal genome structure. Finally, despite multiple studies attempting to study the spatial and temporal dynamics of brain chromatin spatial organization and their implications for gene regulation and relevance for disease [65,67,82], more data and more innovative methods to analyze such data are much needed for the better prioritization and pinpointing of relevant brain regions and developmental stages for brain-related disorders. With more studies examining diverse aspects of 3D genome organization in the brain and other components in the central nervous system (CNS) [32,113,114,115,116], we anticipate novel discoveries for more comprehensively revealing the molecular etiology underlying brain- and CNS-related disorders. 

## 6. Relevant Resources 

There are many software tools developed for chromosome conformation capture (3C)-based data. Here we summarize relevant interactive data visualization tools and exploration platforms in Table 2. A more comprehensive list including computational methods can be found on the 4DN Web Portal https://www.4dnucleome.org/software.html (accessed on 28 February 2022). We also summarize brain-related data sources in Table 3. A collection of references to Hi-C data and papers can be found at https://github.com/mdozmorov/HiC_data (accessed on 28 February 2022). 

## Author Contributions

Conceptualization, Y.L. and M.H.; writing—original draft preparation, W.L., W.Z., M.H. and Y.L.; writing—review and editing, W.L., W.Z., J.C., B.H., M.H. and Y.L.; review, J.C. and B.H.; visualization, W.L., W.Z., J.C. and M.H.; supervision, Y.L. All authors have read and agreed to the published version of the manuscript.

## Figures and Tables

**Table 1:** Summary of imaging- and sequencing-based technologies.

	Imaging-Based	Sequencing-Based
Mapping approach	Absolute spatial coordinates of pre-selected target sequences	Relative spatial relationships among sequencing reads
Sample preparation	In situ hybridization or sequencing needs fixed cells. Live cell measurement possible, e.g., with DAM- or CRISPR-based methods	Lysis needed for sequencing
Multiplicity of contacts	Multiway	Pairwise for 3C-based methods and multiway for ligation-free methods
Spatial distance of detected contacts	Can detect interchromosomal contacts	3C-based methods more often observe intrachromosomal interactions while ligation-free methods also detect abundant interchromosomal contacts
Advantages	Inherently single-cell measurement, preservation of cell location information in the tissue context, direct readout of spatial coordinates, detection of multi-way interactions	High throughput and sequence coverage, no need to preselect loci of interest
Limitations	Limited throughput, or limited resolution when providing genome or chromosome-wide coverage	No direct spatial information, most based on millions of cells, 3C-based interactions are not easily transformed to spatial distance, ligation and fragmentation efficiency, requires high-depth
Representative single-cell technologies	DNA seqFISH+ [99], MERFISH [100], OligoFISSEQ [101], ORCA [102]	Single-nucleus methyl-3C [40], Methyl-HiC [42], Dip-C [36], Nagano et al., 2017 [34], Flyamer et al., 2017 [35], Stevens et al., 2017 [103], sciHi-C [37]

**Table 2:** Software.

Name	Data Type	Description	URL
HUGIn	HiC, PC-HiC, HiChIP/PLAC-Seq	HUGIn is an integrative Hi-C data visualization tool with a built-in database	http://hugin2.genetics.unc.edu (accessed on 28 February 2022)
3D Genome Browser	Hi-C, ChIA-PET, Capture Hi-C, HiChIP/PLAC-Seq	Visualization of the chromosomal contract matrices	http://3dgenome.fsm.northwestern.edu (accessed on 28 February 2022)
WashU Epigenome Browser	5C, Hi-C, ChIA-PET	Supports multiple types of long-range genome interaction data	http://epigenomegateway.wustl.edu (accessed on 28 February 2022)
3DIV	Hi-C	A 3D-genome interaction viewer and database	http://3div.kr (accessed on 28 February 2022)
Juicebox	Hi-C	Software for visualizing data from Hi-C	http://www.aidenlab.org/juicebox (accessed on 28 February 2022)
HiGlass	Hi-C	Displaying and comparing large matrices within a web page	http://higlass.gehlenborglab.org (accessed on 28 February 2022)
Nucleome Browser	Multi-data	Multimodal, interactive data visualization and exploration platform	http://vis.nucleome.org (accessed on 28 February 2022)

**Table 3:** Data Sources.

Species	Tissue/Cell Type	Technology	Reference
Human	Fetal cortical plate and germinal zone	Hi-C	Won et al., 2016 [82]
Human	DLPFC, hippocampus	Hi-C	Schmitt et al., 2016 [11]
Human	Fetal and adult brain	Hi-C	Giusti-Rodriguez et al., 2018 [67]
Human	Brain tissues	Hi-C	Li et al., 2018 [65]
Human	Brain tissues	Hi-C	Wang et al., 2018 [117]
Human	Fetal brain	Capture Hi-C	Song et al., 2019 [61]
Human	Adult brain	PLAC-seq	Nott et al., 2019 [62]
Human	Adult cortex	sc-m3c-seq	Lee et al., 2019 [40]
Mouse	Retina and main olfactory epithelium	Dip-C	Tan et al., 2019 [118]
Mouse	Olfactory sensory neurons	Hi-C	Monahan et al., 2019 [112]
Human	Fetal cortex	PLAC-seq	Song et al., 2020 [63]
Human	Neurogenesis and brain	eHi-C	Lu et al., 2020 [113]
Mouse	Mouse cortical neurons	Hi-C	Beagan et al., 2020 [119]
Mouse	Brain	immuno-GAM	Winick-Ng et al., 2021 [25]
Mouse	Hippocampus	sc-m3c-seq	Liu et al., 2021 [41]
Mouse	Cortex and hippocampus	Dip-C	Tan et al., 2021 [94]
Macaque	Fetal brain	Hi-C	Luo et al., 2021 [120]
Human	Neurons and glia	Hi-C	Hu et al., 2021 [64]
Human	Neural progenitor cells	Hi-C	Rajarajan et al., 2018 [121]
Human	Midbrain dopaminergic neurons	Hi-C	Espeso-Gil et al., 2020 [122]

## Data Availability

No data was reported in this study.

## Abbreviations

Abbreviation	Meaning
1D	One-dimensional
3C	Chromosome conformation capture
3D	Three-dimensional
ABC	Activity-by-contact
AD	Alzheimer’s disease
ADHD	Attention deficit hyperactivity disorder
BD	Bipolar disorder
CNV	Copy number variants
CT	Chromosome territory
DEL	Deletions
DG	Dentate gyrus
DUP	Duplication
FIRE	Frequently interacting region
GWAS	Genome-wide association studies
Hi-C	High-throughput chromatin conformation capture
INV	Inversion
LDSC	Linkage disequilibrium score regression
Mb	Mega-base
MEI	Mobile element insertion
MP	Mental process
PIR	Promoter interacting regions
TAD	Topologically associating domain
sci-Hi-C	Single-cell combinatorial index Hi-C
SCZ	Schizophrenia
scHi-C	Single cell Hi-C
SNP	Single nucleotide polymorphism
SV	Structural variant
UD	Unipolar depression
WES	Whole exome sequencing
WGS	Whole genome sequencing
